# Scleral Micro-RNA Signatures in Adult and Fetal Eyes

**DOI:** 10.1371/journal.pone.0078984

**Published:** 2013-10-21

**Authors:** Ravikanth Metlapally, Pedro Gonzalez, Felicia A. Hawthorne, Khanh-Nhat Tran-Viet, Christine F. Wildsoet, Terri L. Young

**Affiliations:** 1 School of Optometry, University of California, Berkeley, California, United States of America; 2 Duke Eye Center, Duke University Health System, Durham, North Carolina, United States of America; 3 Center for Human Genetics, Duke University, Durham, North Carolina, United States of America; University of Florida, United States of America

## Abstract

**Introduction:**

In human eyes, ocular enlargement/growth reflects active extracellular matrix remodeling of the outer scleral shell. Micro-RNAs are small non-coding RNAs that regulate gene expression by base pairing with target sequences. They serve as nodes of signaling networks. We hypothesized that the sclera, like most tissues, expresses micro-RNAs, some of which modulate genes regulating ocular growth. In this study, the scleral micro-RNA expression profile of rapidly growing human fetal eyes was compared with that of stable adult donor eyes using high-throughput microarray and quantitative PCR analyses.

**Methods:**

Scleral samples from normal human fetal (24 wk) and normal adult donor eyes were obtained (n=4 to 6, each group), and RNA extracted. Genome-wide micro-RNA profiling was performed using the Agilent micro-RNA microarray platform. Micro-RNA target predictions were obtained using Microcosm, TargetScan and PicTar algorithms. TaqMan® micro-RNA assays targeting micro-RNAs showing either highest significance, detection, or fold differences, and collagen specificity, were applied to scleral samples from posterior and peripheral ocular regions (n=7, each group). Microarray data were analyzed using R, and quantitative PCR data with 2^-deltaCt methods.

**Results:**

Human sclera was found to express micro-RNAs, and comparison of microarray results for adult and fetal samples revealed many to be differentially expressed (p<0.01, min p= 6.5x10^11^). Specifically, fetal sclera showed increased expression of mir-214, let-7c, let-7e, mir-103, mir-107, and mir-98 (1.5 to 4 fold changes, p<0.01). However, no significant regionally specific differences .i.e., posterior vs. peripheral sclera, were observed for either adult or fetal samples.

**Conclusion:**

For the first time, micro-RNA expression has been catalogued in human sclera. Some micro-RNAs show age-related differential regulation, higher in the sclera of rapidly growing fetal eyes, consistent with a role in ocular growth regulation. Thus micro-RNAs represent potential targets for ocular growth manipulation, related to myopia and/or other disorders such as scleral ectasia.

## Introduction

A signaling cascade that originates in the retina is believed to direct the scleral changes underlying ocular enlargement or growth [1]. During this process, the sclera undergoes altered extracellular matrix remodeling. In myopia, these changes are exaggerated and ocular elongation accelerated, with the sclera becoming thinner and biomechanically weaker. Because the sclera defines the size and shape of the eye, it is an attractive target for myopia control. To-date studies have linked to “myopic” ocular enlargement altered scleral expression of many genes, including collagens, matrix metalloproteases (MMPs), tissue inhibitors of MMPs, fibroblast growth factor receptor-1, transforming growth factor-beta, and integrins [2,3]. Improved understanding of the mechanisms underlying scleral extracellular matrix remodeling may identify new suitable therapeutic targets for myopia. The purpose of this study was to examine the profile of micro-RNAs within the sclera. 

Micro-RNAs are small non-coding single-stranded RNAs, which serve as key regulators of gene expression at the post-transcriptional level. This regulation is achieved through base pairing with the 3’ untranslated region (UTR) of their target mRNAs, resulting in cleaving and degradation of the mRNAs, either by virtue of perfect or nearly perfect pairing (as seen in plants), or by translational repression resulting from imperfect pairing (as seen in mammals) [4-6]. Micro-RNAs may thus serve as nodes in signaling networks, modulating via gene expression changes, many cell activities including cell proliferation, differentiation, metabolism and apoptosis [7,8]. The potential influence of micro-RNAs on gene expression is immense, with >1000 micro-RNAs already discovered in humans [9], with roles in both normal and disease states [7,8]. 

Micro-RNA transcriptomes have also already been described for some ocular tissues, including the retina, lens, and cornea (for a detailed review, see 10), although the roles of ocular micro-RNAs in normal and disease states remain largely unknown, with a few exceptions. One study implicated four micro-RNAs (mir-96, mir-183, mir-1 and mir-133) in the progression of pathological retinal changes in a transgenic retinitis pigmentosa mouse model [11] and three other micro-RNAs (mir-31, -150 and -184), have been implicated in ocular choroidal neovascularization (CNV, a prominent clinical feature associated with pathological/higher degrees of myopia) [12] and diabetic retinopathy [13]. There is only one report of successful therapeutic targeting of micro-RNAs in ocular disease, involving silencing mir-23 and mir-27 in CNV [14]. Most recently, mutations in the seed region of mir-184 have been shown to cause EDICT (endothelial dystrophy, iris hypoplasia, congenital cataract, and stromal thinning) syndrome and familial keratoconus with cataract [15,16]. 

The current study was based on the premise that the sclera, like most tissues, expresses micro-RNAs, some of which have active roles in modulating the genes regulating scleral matrix remodeling during ocular enlargement. Any also serving as regulators of the scleral gene expression changes linked to myopia would thus represent potential therapeutic targets. The specific aims of this study were to establish genome-wide scleral micro-RNA expression profiles and to identify and validate some potential modulators of accelerated ocular growth (e.g., predicted target micro-RNAs that can potentially regulate collagen gene expression). As a model for the latter, we used normal human fetal eyes, with human adult eyes with stable ocular dimensions serving as our reference model. We used microarray analyses to establish and compare the scleral micro-RNA profiles of these two models. Results for a subset of micro-RNAs identified as having the potential to regulate collagen were validated using quantitative PCR micro-RNA assays in follow-up experiments.

 Although our primary interest is in myopia, obtaining human donor tissue from young eyes with progressing myopia is difficult, if not impossible. Thus fetal eyes were selected as a substitute for the latter in this initial search for key micro-RNA regulators of scleral remodeling during accelerated ocular growth. During gestation, the rate of ocular axial elongation in humans is as high as 0.66 mm/week at 20 weeks and 0.32 mm/week at 30 weeks [17]. A 24-week gestational age was chosen since at this age the sclera closely resembles the adult sclera, both histologically and ultrastructurally, with a fully formed collagen fibril assembly and mature elastin [18]; it is also undergoing active remodeling. Reference tissue came from middle-aged and elderly normal adult subjects, representing the most readily available adult ocular tissue; importantly for our study, their eyes would no longer be undergoing active scleral remodeling. 

In brief, we found that the human sclera expresses many micro-RNAs, some of which show age-related differential expression, consistent with a role in ocular growth regulation. A link between the scleral micro-RNAs confirmed to be up-regulated in fetal sclera and myopia, which also involves rapid ocular enlargement, is plausible but yet to be established.

## Methods

### Donor ocular tissues

Normal human adult eyes (55 to 80 years of age) and normal fetal eyes (24 week gestation) were obtained from the North Carolina Eye Bank and Advanced BioResources (ABR Inc., Oakland, CA), respectively. The study was approved by Duke University’s Institutional Review Board and adhered to the tenets of the Declaration of Helsinki guidelines.

Comprehensive ocular histories were available for adult donor eyes and were reviewed to judge the suitability of the tissue. The histories included surgical history, syndromic connective tissue disorders, and form-deprivation myopia conditions (ptosis, anisometropia, corneal opacities, congenital cataracts, retinopathy of prematurity, etc.), as well as refractive error status and in some cases, ocular axial length. Eyes with posterior segment ocular disease, including glaucoma and age-related macular degeneration, were excluded. Eyes of donors with significant systemic conditions such as cancer, requiring long-term drug therapies with possible effects on the eyes were also excluded. Systemic hypertension and diabetes were not among the exclusion criteria. For all adult donors, the cause of death was given as either heart or pulmonary disease, although their complete medical histories were not always available. Enucleated eyes were received and stored in RNAlater™ within 6 hours of death. In the adult donor group, all eyes were Caucasian and balanced for gender. Fetal donor eyes were collected from elective abortions; none were pre-term and thus not exposed to oxygen. All fetal eyes were harvested within minutes of death for storage in RNAlater™. Fetal samples were only balanced for gender since ethnic information was not available. 

### Dissections and RNA isolation

Although only scleral samples were used in the study described here, retina, RPE-choroid complex and sclera samples were all collected. These tissues were separated by fine dissection from 5 mm trephine punches taken from the posterior pole region that included the macular region, where most of the scleral distension and related retinal thinning occurs in high myopia. An additional, more peripheral punch was collected at least 8 mm away from the fovea, the decision to collect this additional sample being driven by recent interest in the role of the peripheral retina in the regulation of refractive errors and ocular shape [19] [20]. Scleral samples were wiped clean with phosphate-buffered saline, snap frozen in liquid nitrogen, and stored at -80° C until processed. 

In processing scleral samples, they were first homogenized (BeadRuptor, Omni International Inc.) and then total RNA extracted using a miRVANA™ isolation kit (Ambion Inc.), which is designed to preserve the small RNAs. The quality and quantity of RNA were determined using a Nanodrop™ (Thermo Scientific Inc.) and/or an Agilent Nanochip together with a Bioanalyzer (Agilent Inc.) (subunit band clarity and separation, RNA Integrity Number (RIN)). Only samples with A260/A280 ratios of greater than 1.8 and RIN score greater than 7 were further processed.

### Genome-wide scleral micro-RNA expression (micro-RNA microarrays)

Profiling experiments used posterior and peripheral scleral samples from fetal and adult eyes (total 4 groups, n=4 to 6 in each group), combined with Agilent Human micro-RNA microarrays (Rel12.0/16.0; Asuragen Inc.) and high-performance SurePrint Technology™. The arrays were in an 8-plex format (8 arrays per slide), with 60-mer probes and 16-20 replicate features per micro-RNA. A total of 866 (Rel 12.0) or 1205 (Rel 16.0) human micro-RNAs were represented on the microarrays. Micro-RNA profiling experiments were processed at Asuragen Services (Austin,TX), according to standard operating procedures in a GLP-compliant services laboratory. Briefly, total RNA was first dephosphorylated and then the pCp-Cy3 labeling molecule ligated to the 3’ end of the RNA molecules. The labeled RNA was purified using BioSpin6 (Bio-Rad, Hercules CA). Hybridization, washing, staining, imaging, and signal extraction were performed according to Agilent-recommended procedures. 

### Expression of specific scleral micro-RNAs (Taqman micro-RNA Assays – Real-time PCR)

The genome-wide microarray analyses pointed to differential regulation of micro-RNAs between adult and fetal groups. Subsequent validation experiments used TaqMan® micro-RNA assays on posterior and peripheral scleral samples from fetal and adult eyes (total of 4 groups; n=7 in each group) and targeted micro-RNAs showing collagen (Col1A1) specificity (i.e., hsa-let-7b, hsa-mir-214, hsa-let-7c, hsa-let-7e, hsa-mir-103, hsa-mir-107, and hsa-mir-98) and either highest significance, detection, or fold differences in comparisons of equivalent fetal and adult samples in microarray profiling. Micro-RNA target predictions were obtained using Microcosm (http://www.ebi.ac.uk/enright-srv/microcosm/htdocs/targets/v5/), TargetScan (http://www.targetscan.org/) and PicTar (http://pictar.mdc-berlin.de/) algorithms (Scores for target predictions are included in Table S1). Because of the limited amount of samples available, it was not possible to follow-up even the most significant micro-RNAs identified in comparisons of posterior with peripheral samples in microarray analyses. TaqMan® micro-RNA assays contained a stem-looped primer for reverse transcription and a sequence-specific TaqMan® assay (containing forward and reverse primers along with a TaqMan® MGB probe). Reverse transcription reactions specific to the micro-RNAs tested were performed on individual samples, and quantitative PCR reactions undertaken with a starting amount of 5 ng/reaction. RNU44, RNU48, RNU6B, and U47 (all SnoRNAs; small nucleolar RNAs) served as housekeeping genes in these assays. All reactions were run in quadruplicates. 

### Statistics

#### Micro-RNA profiling

The signal processing implemented for the Agilent micro-RNA array was a multi-step process involving probe specific signal detection calls, background correction, and global normalization. For each probe, the contribution of signal due to background was estimated and removed by the Agilent Feature Extraction software as part of the data file output. Detection calls also made use of the Agilent Feature Extraction software. All other analyses were performed in R (http://www.r-project.org). Arrays within a specific experiment were normalized together according to the Variance Stabilization and Normalization (VSN) method described by Huber et al. [21]. The VSN method models the quadratic relationship between the variance of microarray data and the signal intensity, transforming the data such that the variance is roughly constant. This transformation replaces the use of logarithms and has the following properties:

1. comparable to the logarithmic transformation at the high end of the intensity spectrum,
2. roughly linear at lower end of intensity spectrum,
3. valid for negative after-background-subtraction values,
4. results in mild compression of values at lower end of spectrum and, as a result, the differences in VSN transformed values cannot be directly reverted to fold changes.

Principal Components Analysis (PCA) was performed after first normalizing and then mean-centering the data for each probe to check the variance in the dataset. Individual variances are not rescaled in PCA, so that genes with larger variance will have greater weight in determining the principal components than genes with lower variance. Mean expression values, standard deviations, and log_2_ ratios were calculated. For pair-wise comparisons between groups, an ANOVA contrast of the difference between the associated model coefficients was carried out for each micro-RNA, with multiplicity correction applied to the resulting p-values to limit the false discovery rate (FDR) to 0.05. A method described by Benjamini & Hochberg [22] was used to adjust for the overall FDR, allowing the creation of statistically reliable micro-RNA lists. The FDR is defined as the expected value of the ratio of the number of erroneously rejected true hypotheses to the number of rejected hypotheses. Benjamini and Hochberg’s (1995) step-up procedure rejects H(1); : : : ;H(*k*) with *k* being the largest value of *i* for which *p*_*i*_ < *(i*/*m*)*q* in order to control the FDR at level *q* when the *m* distinct p-values *p*_*i*_ are independent. Empirical Bayes methods (as implemented by the LIMMA R package), were used to shrink individual probe/micro-RNA variances towards a common value, also augmenting the degrees of freedom for the individual variance estimates [23]. Both the stability and power of statistical hypothesis testing for individual micro-RNAs were thus enhanced by borrowing information from the entire ensemble of probes. Heatmaps and dendrograms, utilizing Pearson correlation coefficients, have also been generated to illustrate the potential relationship between the expression profiles of the samples (group-wise). To identify the major patterns driving the differences in micro-RNA expression between different groups, the false discovery rate controlled p-values were k-means clustered. The k-means cluster centers were themselves hierarchically clustered to identify the relationships between different clusters.

#### Taqman micro-RNA assays

Quantitative PCR data were analyzed using the Comparative CT method, where the amount of target is normalized to endogenous reference/s, and 2^-deltaCt values and fold differences between groups calculated [24]. The geometric mean of the expression levels of all 4 housekeeping genes was used as the normalization factor [25]. Two-tailed student t-tests were performed, assuming equal variance, and p-values obtained.

## Results

### Micro-RNA profiling

**Figure 1:**
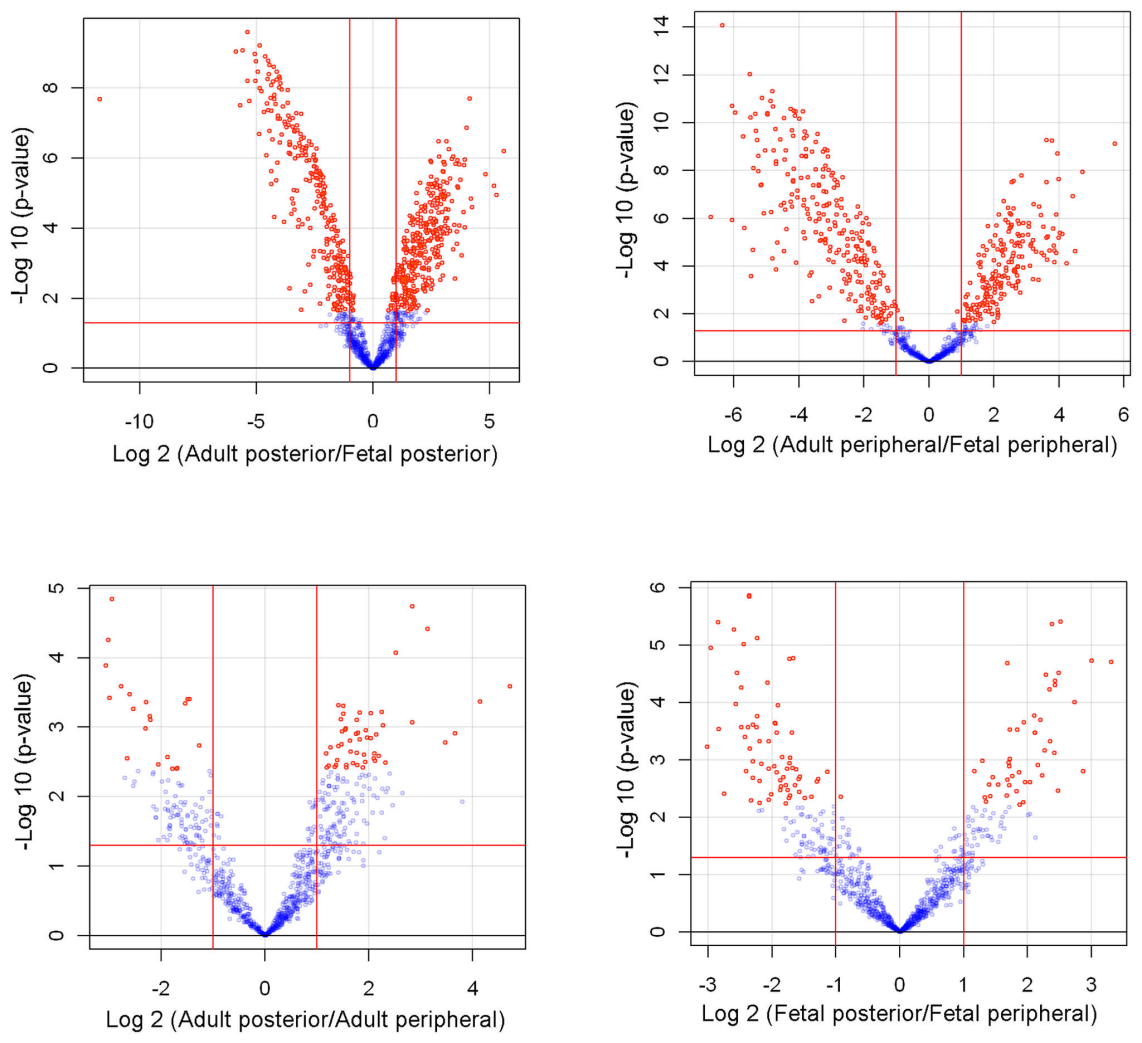
Volcano plots of microarray data showing the relationship between fold change in micro-RNA expression and significance for all pair-wise comparisons (n=4 to 6 per group). The x-axis shows fold change (log_2_ ratio scale) and the y-axis, the negative log_10_ of p-values (higher values indicate greater significances). The two vertical red lines demarcate the area outside of which there is at least a two-fold difference in expression levels between fetal and adult scleras. The single horizontal red line marks the threshold for an unadjusted p-value of 0.05; data points above this line can be considered statistically significant at p-value of 0.05, without correction for multiple testing. Shown in red are data points achieving statistical significance after correction for a 5% false discovery rate (FDR); those not reaching statistical significance are in blue.

A significant number of micro-RNAs showed significant age-related differences in expression, while far fewer micro-RNAs showed regional (ocular) differences in expression within each of the two age groups. Shown in Figure 1 are pair-wise volcano scatter plots comparing results for posterior and peripheral scleral samples from fetal eyes as well as results for equivalent comparisons for adult scleral samples, and comparisons between equivalent samples from fetal and adult eyes. In these plots, the y-axis shows the negative log_10_ of p-values (a higher value indicates greater significance) and the x-axis shows the difference in the intensity values in the log 2 space between the two groups being compared. The data points are color-coded, with red markers signifying micro-RNAs with false discovery rate (FDR) corrected p-value of less than 0.05. 

The comparisons of equivalent adult and fetal samples (Figure 1, top panels), shows a number of micro-RNAs to be differentially expressed to a statistically significant level; two outliers in the adult posterior group were excluded in these analyses. These results contrast with the relatively small number of statistically significant data points in plots comparing posterior versus peripheral samples, for fetal and adult eyes respectively (Figure 1, bottom panels). The mean values of micro-RNA expression as well as the fold difference comparisons across groups along with statistical significance values are provided as supplementary tables (Table S2 and Table S3). The relevant microarray data have also been deposited in NCBI's Gene Expression Omnibus (GEO), and are accessible through GEO Series accession number GSE46435. 

**Figure 2:**
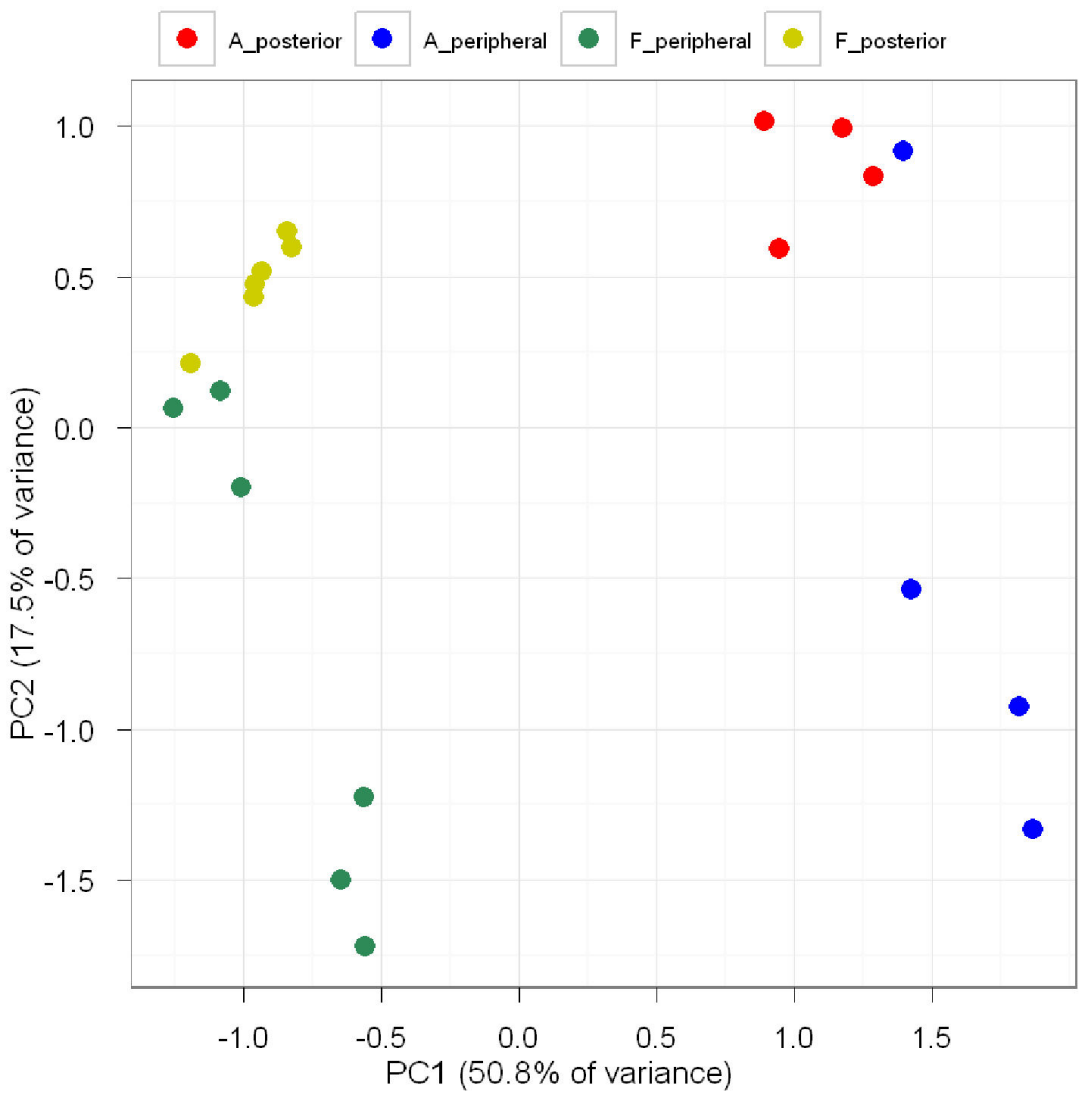
The PCA plot illustrating the correlation of expression between samples. Clustering on the PCA plot indicates a strong correlation. The x-axis shows the first principal component (PC1) revealing variations of expression between ‘adult’ and ‘fetal’ groups. The y-axis shows the second principal component (PC2) revealing variations of expression between ‘posterior’ and ‘peripheral’ groups.

Analysis of the variance in the data provides one way of examining differences between groups, as in the PCA plot shown in Figure 2. The x-axis shows the first principal component (PC1), revealing variations in expression between ‘adult’ and ‘fetal’ groups (50.8%). The y-axis shows the second principal component (PC2), revealing variations in expression between ‘posterior’ and ‘peripheral’ groups (17.5%). Samples from the same group tended to cluster together. The latter trend is also evident in the heatmaps and a dendrogram derived from microarray data (Figure S1). 

**Figure 3:**
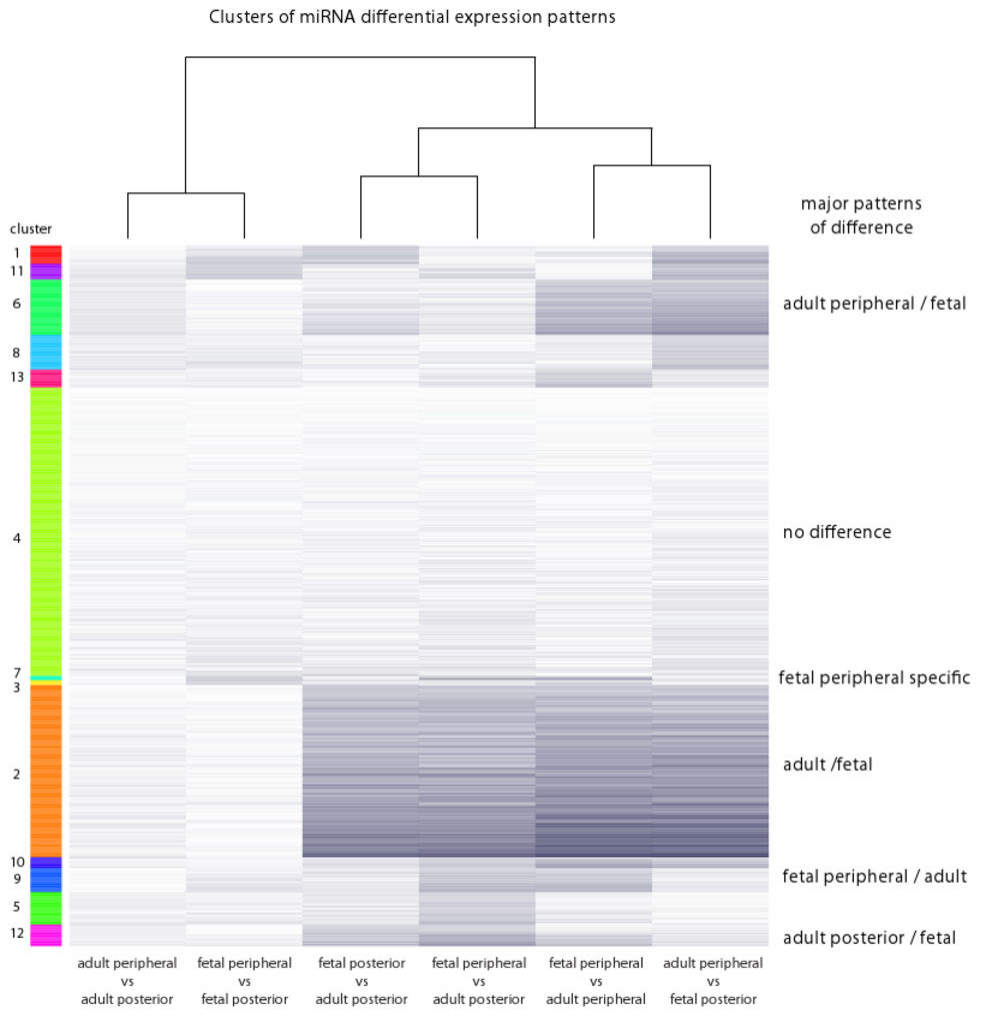
Patterns of micro-RNA expression differences examined by cluster analysis. The false discovery rate controlled p-values were k-means clustered to identify major patterns driving the differences in micro-RNA expression between different samples. The k-means cluster centers were themselves hierarchically clustered to identify the relationships between the different clusters.

Cluster analysis of micro-RNA expression patterns aided in identifying sets of micro-RNAs that were differentially regulated in specific group-wise comparisons (e.g., adult vs. fetal, posterior vs. peripheral etc). From this analysis, it is clear that differences between adult and fetal groups represent the major differences overall. Clusters are depicted in Figure 3 and available as supplementary tables (Table S4). Although the posterior sclera is of most interest in the context of myopia, a number of clusters appear to reflect region-specific differences (e.g., clusters 1, 11, 6, 8, 13 - driven by adult peripheral group; clusters 3, 7, 9 - driven by fetal peripheral group; cluster 12 - driven by adult posterior group). These clusters may be useful in selecting candidates for future investigations aimed at understanding the regulation of ocular shape, and specifically, of the peripheral sclera.

### Taqman micro-RNA assays

**Figure 4:**
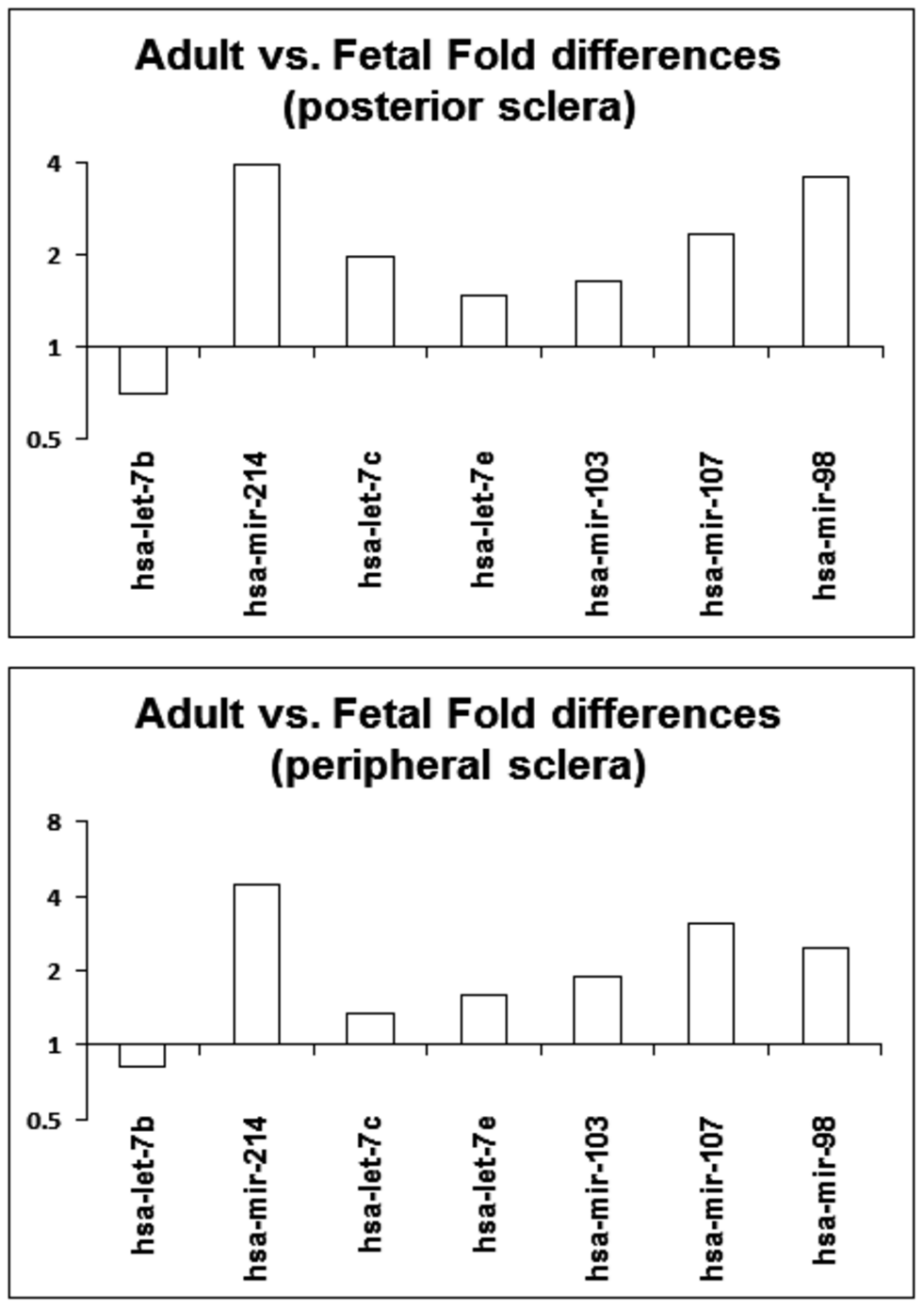
Results from Taqman micro-RNA PCR assays. (Top) Relative fold changes in micro-RNA expression in adult compared to fetal posterior sclera (n=7); all micro-RNAs except miR-let7-b were significantly up-regulated in fetal compared to adult samples. (Bottom) Relative fold changes in micro-RNA expression in adult compared to fetal peripheral sclera (n=7); all micro-RNAs except hsa-let7b and hsa-let7c were significantly up-regulated in fetal compared to adult samples. (Y-axis in log scale to the base 2).

That many scleral micro-RNAs show significant, age-related differential expression, as revealed in microarray experiments, was confirmed with quantitative PCR assays, which used a slightly expanded cohort (n=7 posterior and peripheral samples for both fetal and adult eyes). Figure 4 shows the results of comparisons of equivalent fetal and adult samples, expressed as relative fold differences for the selected micro-RNAs – hsa-let-7b, hsa-mir-214, hsa-let-7c, hsa-let-7e, hsa-mir-103, hsa-mir-107, and hsa-mir-98; microarray data are also shown for reference. In the posterior sclera, all micro-RNAs (except hsa-let7b) were significantly up-regulated in fetal compared to adult samples (Figure 4), while in the peripheral sclera, all selected micro-RNAs except hsa-let7b and hsa-let7c were significantly up-regulated in fetal compared to adult samples (Figure 4). For the same set of micro-RNAs, comparisons of micro-RNA expression in posterior compared to peripheral sclera within each of the age groups did not reveal any significant differences. Table 1 lists for both age groups, the average 2^-deltaCt values and their standard deviations (SD) from which the fold differences were calculated, along with significance (p) values and corresponding microarray findings. Since the “fold difference” parameter is derived from ratio data, the 2^-deltaCt SDs is considered a better index of the variability in the data. In general, the quantitative PCR findings were in agreement with the microarray findings.

**Table 1:** Mean 2^-deltaCt values and corresponding standard deviations (SD) for posterior and peripheral scleral samples from adult and fetal eyes; adult versus fetal eye fold differences and related P-values listed for the same samples.

**micro-RNAs**	**2^-deltaCt for adult eyes**		**2^-deltaCt for fetal eyes**		**Fold differences (QPCR)(adult vs fetal)**	**P-value**	
	**Mean**	**SD**	**Mean**	**SD**			**Fold differences (microarray) (adult vs fetal)**
	**Posterior sclera**						
hsa-let-7b	13.28	3.03	9.32	1.33	0.7	*0.008*	-0.17
hsa-mir-214	2.46	0.92	9.81	1.87	3.98	*7.93* *E-07*	-2.41
hsa-let-7c	5.77	1.69	11.5	2.17	1.99	*0.0001*	-2.00
hsa-let-7e	14.77	6.42	21.79	3.55	1.47	*0.02*	-2.66
hsa-mir-103	1.44	0.37	2.38	0.35	1.64	*0.0004*	-1.73
hsa-mir-107	0.03	0.01	0.08	0.01	2.34	*1.96* *E-05*	-1.85
hsa-mir-98	0.1	0.04	0.38	0.07	3.6	*1.46* *E-06*	-2.13
	**Peripheral sclera**						
hsa-let-7b	13.76	3.61	11.11	2.91	0.8	0.19	-1.62
hsa-mir-214	2.69	0.7	11.98	0.99	4.44	*1.45* *E-06*	-3.26
hsa-let-7c	6.55	2.76	8.78	2.06	1.34	0.11	-2.84
hsa-let-7e	15.32	7.94	24.22	6.38	1.58	*0.03*	-4.16
hsa-mir-103	1.35	0.64	2.53	0.83	1.87	*0.01*	-3.14
hsa-mir-107	0.03	0.01	0.09	0.05	3.13	*0.007*	-2.83
hsa-mir-98	0.11	0.07	0.29	0.05	2.47	*0.0006*	-3.19

Upregulation in the fetal group is indicated by values greater than 1 for QPCR data, and negative values for microarray data.

## Discussion

Our study represents the first comprehensive study of micro-RNA expression in the sclera. We not only established a catalogue of micro-RNAs for the human sclera, but in comparing scleral expression profiles of fetal and adult human eyes, we also identified a subset of micro-RNAs that could potentially be involved in ocular growth regulation. The findings of this study are discussed in the context of matrix remodeling and potential roles for micro-RNAs in abnormal ocular growth, such as myopia, which is our primary research interest.

Our study revealed significant differences in the scleral micro-RNA expression profile of fetal eyes compared to that of adult eyes. Because fetal eyes are rapidly growing while the eyes of older adults are likely to be relatively stable in their overall dimensions, we rationalized that some of the identified differentially expressed micro-RNAs may be involved in modulating gene expression critical to ocular growth regulation. Furthermore, since the rate of ocular expansion primarily reflects scleral collagen regulation and so extracellular matrix remodeling, focusing our efforts on micro-RNAs known to be involved collagen gene regulation would seem most likely to capture those involved in ocular growth regulation. 

 To-date, a number of micro-RNA families have been implicated in collagen gene regulation in cardiovascular, liver, muscle, renal and eye diseases (specifically glaucoma) [26-30], with the mir-29 family being most consistently implicated. It is thus of interest that our scleral microarray analyses revealed age-related differential expression of miR29b (i.e., miR29b, adult vs. fetal – posterior: 2.2 fold, p<0.01; peripheral: 2.6 fold, p<0.01), although since the expression differences were relatively modest compared to those of many other micro-RNAs analyzed in our microarray, mir-29b was not selected for follow-up validation experiments. 

Of the seven micro-RNAs selected for follow-up quantitative PCR analysis from the larger dataset generated from our microarray analyses (hsa-let-7b, hsa-mir-214, hsa-let-7c, hsa-let-7e, hsa-mir-103, hsa-mir-107, & hsa-mir-98), all but two of these micro-RNAs (hsa-let7b and hsa-let7c) showed significant up-regulation in fetal compared to adult sclera, irrespective of whether tissue samples were collected from the posterior pole or more peripherally. In the case of hsa-let7b and hsa-let7c, only posterior scleral samples showed significant differences in expression between fetal and adult eyes. Noteworthy perhaps is that the micro-RNA let-7 family has recently been implicated in collagen modulation in pancreatic cancer cells [31]. Future studies directed at underlying signal pathways and mechanisms will follow-up on the possible involvement of the let-7 family in scleral collagen remodeling. 

Relatively little is known about the functions in gene regulation of micro-RNAs (hsa-mir-214, hsa-mir-103, hsa-mir-107, & hsa-mir-98), although one member of this group, mir-214, has been implicated in cell survival in cancer [32] and another, mir-107, has been linked to Alzheimer’s disease pathogenesis [33]. However there is no obvious link between the latter observations and extracellular matrix remodeling. Nonetheless, because of the predicted collagen binding affinity of these micro-RNAs, roles in ocular growth regulation via scleral remodeling are possible. Another micro-RNA, mir-328, showed a modest difference in expression (1.5 fold, p<0.01) in peripheral fetal compared to adult sclera in our microarray analyses. While this finding was not validated by PCR, the possible involvement of mir-328 in myopia development via the regulation of the PAX6 gene and retinoic acid pathway in RPE cells is suggested in a recent *in vitro* study by Chen et al. [34].

As mentioned previously, working within the limitations of ocular donor tissues, we made every possible effort to control for variables unrelated to ocular growth that could confound our results. Nonetheless, we must also acknowledge three potentially important limitations of our studies: 1) the lack of systemic medical histories of some adult donors, 2) the use of differences in donor age as a surrogate for differences in ocular growth rates, and 3) potential sources of micro-RNAs in the sclera other than fibroblasts. In relation to the first point, we believe that undocumented systemic diseases are not likely to have biased our results on the basis that it is unlikely that systemic disease conditions were significantly over-represented in donors with incomplete records. Furthermore, any increase in the noise in our data level, a more likely consequence of undocumented systemic disease, would have reduced our ability to detect expression differences related to age alone. In relation to the second point, it is possible that the mechanisms underlying rapid, albeit normal ocular expansion during fetal development are not synonymous with those underlying the accelerated ocular expansion underlying myopia. It is also possible that additional factors, apart from the high rate of ocular growth of fetal eyes, are reflected in their scleral micro-RNA profiles. Nonetheless, the identified subset of scleral micro-RNAs, linked to both rapid normal ocular growth and collagen gene regulation, would seem a reasonable starting point for investigations aiming to identify scleral micro-RNAs involved in “myopic” growth. That the study yielded a catalogue of micro-RNAs for fetal and adult human sclera also represents a valuable resource beyond myopia, given the increasing interest in this tissue in relation to glaucoma, among other ocular diseases [35,36]. Addressing the third point, extracellular matrix producing fibroblasts are the predominant cells in the sclera although there may be other cells such as melanocytes and transient inflammatory response cells (unlikely in our samples) [37,38]. The contribution from vascular elements is also likely to have been minimal, given that the sclera is essentially avascular except for localized blood vessels traversing it on route to internal ocular structures, both near the optic nerve and at the equator of the eye [37]. The optic nerve, which includes the largest of these vessels, was avoided in collecting our samples. Furthermore, there are several studies that have examined micro-RNA expression in fibroblasts and their potential role in disease and matrix remodeling [31,39-42]. While it is premature to speculate on the specific roles of micro-RNAs in scleral function, they warrant further investigation and have immense potential both as gene regulators and drug targets. Studies aimed at understanding related mechanisms are an obvious first step.

In summary, this is the first comprehensive study of micro-RNA expression in human sclera. The sclera was found to express micro-RNAs, some of which show age-related differential regulation, higher in rapidly growing fetal eyes, consistent with a potential role in ocular growth regulation. A role for these scleral micro-RNAs in the development of myopia, which also involves accelerated eye growth, is thus plausible. The current findings provide a platform for new lines of mechanistic/pathway-related investigations directed at understanding the role of micro-RNAs in scleral remodeling. Understanding the role of micro-RNAs in scleral mechanisms underlying myopia may open up new anti-myopia treatment strategies. 

## Supporting Information

Table S1 **micro-RNA predictions for Col1a1.**
(XLS)

Table S2 **Summarized values.**
(XLSX)

Table S3 **Gene expression differences.**
(XLSX)

Table S4 **Clusters.**
(XLSX)

Figure S1 **Pearson correlation.**
(TIF)
